# Impact of Supplementary Microbial Additives Producing Antimicrobial Substances and Digestive Enzymes on Growth Performance, Blood Metabolites, and Fecal Microflora of Weaning Pigs

**DOI:** 10.3390/ani11051217

**Published:** 2021-04-23

**Authors:** Hyuk-Jun Lee, Hyeon-Tak Noh, Dimas Hand Vidya Paradhipta, Young-Ho Joo, Seong-Shin Lee, Jeong-Seok Choi, Dong-Hyeon Kim, Soo-Ki Kim, Sam-Churl Kim

**Affiliations:** 1 Division of Applied Life Science (BK21Plus, Institute of Agriculture and Life Science), Gyeongsang National University, Jinju 52828, Korea; hyukjun0209@gmail.com (H.-J.L.); nht1647@gmail.com (H.-T.N.); dimazhand@gmail.com (D.H.V.P.); wn5886@gmail.com (Y.-H.J.); seongshin73@gmail.com (S.-S.L.); x47677104@gmail.com (J.-S.C.); kimdh3465@gmail.com (D.-H.K.); 2 Department of Animal Nutrition and Science, Faculty of Animal Science, Universitas Gadjah Mada, Yogyakarta 55281, Indonesia; 3 Department of Animal Science and Technology, Konkuk University, Seoul 05029, Korea; sookikim@konkuk.ac.kr

**Keywords:** dual-purpose additive, immune response, pathogenic bacteria, probiotics, weaning pig

## Abstract

**Simple Summary:**

The aim of the present study was to confirm that microbial additives producing antimicrobial substances and digestive enzymes increased the health of weaning pigs by improving blood metabolites and fecal microflora. *Pediococcus acidilatic* BBG-L1 and *Lactobacillus plantarum* SK3121 produced antimicrobial activity, while Bacillus subtilis SK877, B. subtilis BBG-B20, and *Saccharomyces cerevisiae* BBG-Y6 produced digestive-enzyme activity. The mixtures of these microbes were used as microbial additives and applied into weaning pigs for 21 d with different levels following 0, 0.5, 1.0, and 1.5% of the diet as fed. In the present study, dietary microbial additives had no effects on growth performance of weaning pigs, except for the feed efficiency. However, dietary microbial additives could improve the health status of weaning pigs. This could be seen in increasing immune response, glucose, and insulin in the blood, as well as reducing *Salmonella* and *Escherichia coli* in the fecal samples of the pigs. Furthermore, the supplementary microbial additive at 1.0% presented the highest improvement in blood metabolites. Therefore, the present study concluded that dietary microbial additives presented antifungal and digestive-enzyme activities that improved the health status of weaning pigs, and a supplementary level of 1.0% was recommended to improve feed efficiency, blood metabolites, and fecal microflora effectively.

**Abstract:**

The present study investigated the effects of microbial additives producing antimicrobial and digestive-enzyme activities on the growth performance, blood metabolites, and fecal microflora of weaning pigs from 21 to 42 d of age. A total of 144 weaning pigs (1:1 ratio of gilt and boar; 21 d of age; 7.40 ± 0.53 kg of average body weight) were randomly distributed into four supplementary levels of microbial additive (0 vs. 0.5 vs. 1.0 vs. 1.5% of fresh weight) with three pens of replication, consisting of 12 weaning pigs per pen. All weaning pigs were maintained with the same basal diet for 21 d. Blood and feces were subsampled at day 21. Feed efficiency tended to increase linearly (*p* = 0.069) with an increasing supplementation level. Insulin, insulin-like growth factor 1, and blood glucose presented a quadratic effect (*p* < 0.05) with an increasing supplementation level, and these blood metabolites were highest at the 1% supplementation level. Immunoglobulin G in blood increased linearly by (*p* < 0.05) increasing the supplementation level. *Salmonella* and *Escherichia coli* in feces were decreased linearly by (*p* < 0.05) increasing the supplementation level. In conclusion, supplementation of microbial additive at 1.0% improved the feed efficiency, blood metabolites, and fecal microflora of weaning pigs.

## 1. Introduction

Weaning pigs are very susceptible to environmental stress and gastrointestinal disorders due to the inflammation caused by pathogenic bacteria such as *Salmonella* and *Escherichia coli* [1,2]. In previous decades, antibiotic growth promoters have been used therapeutically on farms to improve the growth performance, health, and well-being of weaning pigs, and for the prevention of diseases [3]. Even though they have presented many beneficial effects for these animals, the use of antibiotics must be reduced due to the development of resistance by several strains of pathogenic bacteria [2,3]. Nowadays, many studies are seeking to develop a feeding strategy to reduce and minimize the use of antibiotics in weaning pigs. The alternatives to antibiotics, such as probiotics, promise a similar beneficial effect to antibiotics in weaning pigs [1,2,4,5]. Previous studies found that application of probiotics could reduce inflammation by pathogenic bacteria in the gut of weaning pigs, and then increase the pigs’ performance and health [2,3,4]. Moreover, the probiotics could promote the development of the intestinal tract in weaning pigs [6].

In general, three main cultured microbes are commonly used as additives in pigs: lactic acid bacteria (LAB), *Bacillus*, and yeast [2,5,7]. Several strains of cultured LAB and *Bacillus* are able to produce antimicrobial substances [8,9], which can help to improve the performance of weaning pigs. Additionally, strains of cultured *Bacillus* or yeast were also reported to produce digestive enzymes [10,11]. Selected microbes consisting of LAB (*Pediococcus acidilactic* and *Lactobacillus plantarum*), *Bacillus subtilis*, and *Saccharomyces cerevisiae* were isolated from different sources and selected because of their ability to produce antimicrobial substances or digestive enzymes such as protease, amylase, and lipase. A mixture of these microbes as a feed additive was reported to improve the meat quality of Korean native chicken and Hanwoo beef cattle [12,13]. However, the effects of these microbes on weaning pigs have not been tested yet. In the present study, the mixture of these selected microbes was applied to weaning pigs as a microbial additive to study the dual antimicrobial and digestive-enzyme activities. Antimicrobial activity might have beneficial effects by improving immune response and decreasing inflammation of the gut by pathogenic bacteria. On the other hand, the digestive activity might have a positive effect by increasing nutrient absorption in the intestinal tract. In our hypothesis, dietary microbial additives are expected to produce dual activities to improve not only growth performance and blood metabolites, but also fecal microflora in weaning pigs. Therefore, the present study aimed to investigate the effects of microbial additives producing antimicrobial and digestive-enzyme activity on the growth performance, blood metabolites, and fecal microflora of weaning pigs.

## 2. Materials and Methods

### 2.1. Microbial Preparation

The microbial additives in the present experiment were obtained in dry form from Bigbiogen Co., Ltd. (Anseong, Gyeonggi Province, Korea), and consisted of *P. acidilactic* BBG-L1, *Lactobacillus plantarum* SK3121, *B. subtilis* SK877, *B. subtilis* BBG-B20, and S*. cerevisiae* BBG-Y6. The profiles of microbial additives in the present study are presented in Table 1.

Before being applied to the animals, the actual counts of LAB, *Bacillus*, and yeast on microbial additive were enumerated in the present study. Twenty grams of microbial additive were blended with 180 mL of distilled water for 30 s and filtered through two layers of cheesecloth for extraction [14,15]. Microbial counts were measured using an extraction (first dilution) that was continued into several dilutions (10^−6^ to 10^−8^). The LAB count used lactobacilli and de Man, Rogosa, Sharpe (MRS) agar (Difco, Detroit, MI, USA), the *Bacillus* count used Luria-Bertani (LB) agar (Difco, Detroit, MI, USA), and the yeast count used potato dextrose agar (PDA; Difco, Detroit, MI, USA). The MRS agar plates were placed in a CO_2_ incubator (Thermo Scientific, Waltham, MA, USA) at 30 °C for 48 h. The LB agar and PDA plates were incubated for 48 h at 30 °C in an aerobic incubator (Johnsam Corp., Boocheon, Gyeonggi Province, Korea) [16]. Visible colonies from the plates were calculated and the number of colonies forming units (cfu) was expressed per gram of fresh weight. The microbiological data were transformed to log10.

### 2.2. Animals and Management

A total of 144 weaning pigs with a 1:1 ratio of gilt and boar (Landrace × Yorkshire × Duroc; 21 d of age; and 7.40 ± 0.53 kg of average body weight) were randomly distributed into four treatments with three pens of replication, consisting of 12 weaning pigs per pen, which followed the animal-management system in the farm. The present study considered a real-farm situation, for which each treatment was designed with a low number of pens but a high number of animals. The three experimental treatments were as follows: 0% (basal diet without supplementation), 0.5% (basal diet with microbial-additive supplementation of 0.5%), 1.0% (basal diet with microbial-additive supplementation of 1%), and 1.5% (basal diet with microbial-additive supplementation of 1.5%). In addition, 25 kg of basal diet was prepared for each pen and then mixed with microbial additive following each treatment. The basal diet was formulated to meet the nutrient requirements of growing pigs according to the Korea Feeding Standards for Swine [17]. All ingredients of the basal diet are presented at Table 2. The feeding trial was conducted for 21 d, with 7 d of adaptation period previously. The piglets were weaned and housed in pens with automatically controlled light and temperature, and fully slatted floors with concrete or plastic panels. Each pen (1.8 m × 1.8 m) was equipped with a one-hole feeder and nipple waterer to provide diets and water that were available ad libitum. Piglets were fed twice a day at 9:00 and 17:00. During the 21 days, diet, refusal, and fecal samples were collected daily to measure the feed intake and feed efficiency of each pen. For analysis of microflora populations, fecal samples from weaning pigs were collected before (day 0) and at the end of the feeding trial (day 21).

### 2.3. Laboratory Analysis

#### 2.3.1. Chemical Composition of Diets

The diets (1 kg) were dried at 65 ℃ for 48 h and ground to pass through a 1 mm screen using a cutting mill (Shinmyung Electric Co., Ltd., Gimpo, Gyeonggi Province, Korea) according to the protocol of several previous studies [14,15]. The dry-matter (DM) concentration was analyzed using a forced-air drying oven at 105 ℃ for 24 h. The total ash (CA) was determined by incineration in a muffle furnace at 550 ℃ for 4 h. The crude protein (CP) and ether extract (EE) contents were measured by Kjeldahl (method number 984.13 of AOAC [18]) and Soxhlet (method number 920.39 of AOAC [18]) methods, respectively. The digestible energy was determined by calculating the amount of energy in the feed and in the feces. The amount energy in both feed and feces was measured using a bomb calorimeter (Parr 6100; Parr Instrument, Moline, IL, USA). The calcium and phosphorous were analyzed using the wet-ash method (method number 935.15 of AOAC [18]).

#### 2.3.2. Growth Performance

For the analysis of growth performance, each piglet was weighed before and at the end of the feeding trial to calculate the average daily gain (ADG). Feed intake was measured per pen, and then calculated individually to obtain the average daily feed intake (ADFI). Feed efficiency was determined by dividing ADG by ADFI over a period of 21 d (gain/intake).

#### 2.3.3. Blood Metabolites

At day 21, all piglets were bled from the jugular vein using 10 mL vacuum tubes containing K3EDTA (Becton Dickinson, Franklin Lakes, NJ, USA), and the samples were centrifuged at 3000× *g* for 15 min to separate the serum. All assays for blood metabolites (immunoglobulin G (IgG), insulin, insulin-like growth factor 1 (IGF-1), blood urea nitrogen (BUN), and glucose) were performed by Green Cross (GC Pharma Corp., Youngin, Gyeonggi Province, Korea). The serum IgG levels were determined using commercial enzyme-linked immunosorbent assay (ELISA) kits. The concentrations of IGF-1 were determined using the IMMULITE 2000 system (Siemens, Malvern, PA, USA) following the chemiluminescence immunoassay (CIA) technique. The concentrations of insulin and BUN were determined using a commercial radioimmunoassay (RIA) kit (Roche, Mannheim, Germany) and a Urea/BUN kit (Roche, Mannheim, Germany), respectively. An enzymatic kinetic assay was used to determine blood glucose (GLU kit; Roche, Mannheim, Germany). The sensitivity of assays were: 1.5 ng/mL, 1 ng/mL, 14 g/mL, 1 ng/mL, and 1 ng/mL for IgG, insulin, IGF-1, BUN, and blood glucose, respectively. The intra- and interassay CVs were 4.07% and 6.90%, 9.9% and 3.7%, 3.1% and 7.5%, 6.3% and 9.1%, and 1.1% and 1.2%, respectively for IgG, insulin, IGF-1, BUN, and blood glucose.

#### 2.3.4. Fecal Microflora

Fecal samples (200 g) were collected weekly from five spots of each pen and immediately analyzed to measure LAB, *Salmonella*, and *Escherichia coli* counts. Ten grams of each fecal sample was weighed and placed into a stomacher bag with 100 mL of sterile saline (0.9%) at a dilution of 1:10. The protocol for counting LAB was as previously explained. The *Salmonella* count used *Salmonella Shigella* (SS) agar (Difco, Detroit, MI, USA), and the *E. coli* count used Violet Red Bile agar (Difco, Detroit, MI, USA). The SS agar and Violet Red Bile agar plates were incubated for 48 h at 37 °C in an aerobic incubator (Johnsam Corp., Boocheon, Gyeonggi Province, Korea). Visible colonies from the plates were calculated, and the number of cfu was expressed per gram of fecal extract at day 0 and day 21 of the feeding trial. The microbiological data were transformed to log10.

### 2.4. Statistical Analysis

All data were analyzed using the polynomial contrast procedure of the Statistical Analysis System (SAS, Version 9; Cary, NC, USA) [19]. Orthogonal coefficients for linear (L), quadratic (Q), and cubic (C) contrast were adjusted to account for the supplementation levels using the Interactive Matrix Programming Language (PROC IML) procedure of SAS. Then, the General Linear Model (PROC GLM) of SAS was used to examine linear, quadratic, and cubic effects of increasing the supplementation level. A Tukey’s test was used to identify differences among treatments. Significance was declared at *p* ≤ 0.05, while tendency was considered at 0.05 ≤ *p* ≤ 0.10.

## 3. Results

### 3.1. Chemical Compositions of Basal Diet

The basal diet in the present study had digestible energy of approximately 3500 kcal/kg (Table 2). The DM, CP, EE, and CA of the basal diet were 86.8, 21.2, 7.37, and 5.17%, respectively. The calcium and phosphorous of the basal diet were 0.65 and 0.80%, respectively.

### 3.2. Microbial Count of Additives

In the actual count before being applied to the animals, the microbial additives contained LAB at 8.30 log10 cfu/g, Bacillus at 8.94 log10 cfu/g, and yeast at 8.59 log10 cfu/g (Table 3).

### 3.3. Growth Performances

The feed efficiency of weaning pigs tended to increase linearly (*p* = 0.069) with an increased supplementation level of microbial additives in the basal diet (Table 4). Generally, the supplementary level of microbial additives at 1.0% had higher (*p* < 0.05; 0.73 vs. 0.68, 0.69, and 0.70) feed efficiency than at 0, 0.5, and 1.5%. The initial body weight, final body weight, ADFI, and ADG were not affected by the dietary microbial additives.

### 3.4. Blood Metabolites

Concentrations of insulin (*p* = 0.034), IGF-1 (*p* = 0.043), and glucose (*p* = 0.001) in the blood increased quadratically with an increased supplementation level of microbial additives in the basal diet (Table 5). Generally, the supplementary level of microbial additives at 1.0% had higher insulin (*p* < 0.05; 0.64 vs. 0.28, 0.42, and 0.46 μU/mL) and blood glucose (*p* < 0.05; 123.2 vs. 99.9, 103.8, and 105.9 mg/dL) than at 0, 0.5, and 1.5%. The BUN concentration in the blood was not affected by the dietary microbial additives.

### 3.5. Fecal Microflora

Fecal microflora consisting of LAB, *Salmonella,* and *E. coli* were similar among treatment at day 0 of the feeding period (Table 6). At day 21 of the feeding period, LAB tended to increase linearly (*p* = 0.064) with an increased supplementation level of microbial additives in the basal diet, while *Salmonella* (*p* = 0.033) and *E. coli* (*p* = 0.048) decreased linearly.

## 4. Discussion

The actual counts of LAB were reported to be lower than the original counts, while the counts of *Bacillus* and yeast were similar to the original counts. The decreases in LAB counts could have occurred normally due to the storage time. Nevertheless, the actual counts of LAB, *Bacillus*, and yeast were in the recommended range as feed additives for animals according to a previous study, which found such range to be 10^6^ to 10^8^ cfu/g [2]. In the present study, the initial body weight was not different statistically among treatments, which indicated a similar condition of the piglets when the experiment was started. Generally, the growth performances of weaning pigs were not affected by supplementary microbial additives, but a supplementation level of 1.0% tended to result in the highest feed efficiency. Similar to the present study, Suo et al. [5] reported that supplementary microbial additives presented a quadratic pattern for feed efficiency of pigs according to the doses of application. According to Pan et al. [20], microbial additives are capable of enhancing immune responses and attenuating intestinal damage, thus improving weaning pig performance. Moreover, microbial additives were reported to have beneficial and antibiotic effects. This indicated that microbial additives were an alternative feed additive to replace the use of antibiotics. Choi et al. [21] reported that dietary microbial additives containing a mixture of *L. acidophilus*, *B. subtilis*, and *S. cerevisiae* improved ADG, ADFI, and feed efficiency of weaning pigs. In the study, dietary yeast culture as an additive also was effective in increasing digestibility and growth performance of weaning pigs [22]. The growth performances of weaning pigs increased with supplementation of a single LAB, such as *L. acidophilus,* in diet [23]. In contrast, Nguyen et al. [7] reported that a microbial additive containing a mixture of *Bacillus* spp. had no effects on growth performance of weaning pigs, but improved fecal bacteria due to the presence of antimicrobial substance from *Bacillus* spp. Similar to Nguyen et al. [7], Xuan et al. [24] also reported that microbial additives containing *S. cerevisiae* and *Bacillus* spp. had no effects on growth performance of weaning pigs. Based on those previous studies, effects of microbial additives on growth performances of weaning pigs could be varied depending on the microbe species and strain. There are several factors that affect the effectiveness of microbial additives to improve the growth performance of pigs, including microbial strains, doses, environment, physiological condition, and duration of treatment [2,25].

The concentration of IgG in blood could reflect the immune response of the animal, with a higher IgG concentration in blood indicating a better immune response. Liu et al. [26] reported that dietary direct-fed microbes, prebiotics, yeast, or plant extract potentially could improve the immune response of pigs, even though the result might not be consistent in every trial, depending on the animal condition, doses, and environment. The results for IgG in the present study were in agreement with previous studies that reported a similar improvement of IgG by using supplementary microbial additives [6,27]. In addition, this result supported the microbial count in feces, in which the populations of *Salmonella* and *E. coli* were reported to decrease linearly with an increasing supplementation level (Table 6). The mechanism of microbial additives to enhance immune response is not fully understood, but their ability to modify the microbial ecosystem in the gut might improve the immune response of weaning pig [6,26].

Supporting the results for feed efficiency, supplementary microbial additives at 0.1% improved insulin and glucose concentrations in the blood. The increased concentrations of insulin and glucose in the blood might be a response to increased energy absorption in the intestine [2,5,25]. Microbial additives were reported to increase the length of intestinal villi, which increased the nutrient-absorption surface in the small intestine and apparent digestibility [2,5,25]. The other reason for higher glucose and insulin could be due to the activity of digestive enzymes from *B. subtilis* SK877, *B. subtilis* BBG-B20, and *S. cerevisiae* BBG-Y6, which increased the nutrient digestibility of weaning pigs. Antimicrobial activity produced by *P. acidilactic* BBG-L1 and *L. plantarum* SK3121 could also have a role in effectively inhibiting pathogenic bacteria in the digestive tract to assist in better absorption of nutrients by weaning pigs, according to Liao and Nyachoti [2]. On the other hand, IGF-1 concentration in blood had a positive correlation with the growth performance of weaning pigs [28]. IGF-1 was highest with supplementation at 1.0%, which supported the results of insulin, blood glucose, and feed efficiency. However, supplementation at 1.5% had similar results to 0% and 0.5% on insulin, IGF-1, and blood glucose, which indicated a saturated condition when it was supplemented at a high dose. In some cases, an overdose of microbial additives can even reduce immune response and decrease growth performance [5,25,26,29]. The high concentration of BUN in blood reflected the excretion of nitrogen by the animals, which indicated a low utilization of nitrogen for protein synthesis [30,31]. Devi and Kim [32] reported that supplementary *Enterococcus faecium* had no effect on BUN concentration in the blood of weaning pigs, while Liu et al. [33] reported that supplementary *L. brevis* decreased BUN concentration. The reason for the increase of BUN concentration at higher supplementation levels of microbial additives was unclear in the present study. It might have been caused by higher absorption of nutrients, including nitrogen, at higher supplementation levels of microbial additives, which would support the results for feed efficiency.

A population of complex microbes in feces could indicate such population in the gut. In fecal microflora, increasing the supplementation level of microbial additives increased the LAB count, but decreased *Salmonella* and *E. coli* counts linearly after 21 d of feeding due to the presence of antimicrobial activity by *P. acidilactic* BBG-L1 and *L. plantarum* SK3121. These results were in agreement with several previous studies [4,7,23,34], which also reported a beneficial effect of microbial additives to inhibit pathogenic bacteria in feces. A population of pathogenic bacteria in feces has a positive correlation with their population in the gut. High populations of *Salmonella* and *E. coli* in the gut of weaning pigs reduce nutrient absorption for the host and cause gastrointestinal disorders such as diarrhea [25]. With a decreased population of pathogenic bacteria, it can improve gut health and reduce diarrhea frequency in weaning pigs [2,26].

## 5. Conclusions

In general, supplementary microbial additives presented beneficial effects on weaning pigs. It could be seen that microbial additives presented digestive enzyme activity by improving blood metabolites and antimicrobial activity by improving fecal microflora in weaning pigs. The fecal microflora of weaning pigs also improved with higher supplementation levels of microbial additives. The present study reported that supplementary microbial additives at 1% presented better feed efficiency, insulin, and blood glucose than the other supplementation levels. Therefore, we concluded that the selected microbial additives used in the present study should be recommended at 1% supplementation to improve growth performance, blood metabolites, and fecal microflora of weaning pigs.

## Figures and Tables

**Table 1:** Isolation sources, characteristics, and microbial counts of the additives in the present study.

Species	Sources	Characteristics	Cfu ^2^
*Pediococcus acidilactic* BBG-L1	Chick fecal	Antimicrobial activity	1 × 10^9^
*Lactobacillus plantarum* SK3121	Kimchi ^1^	Antimicrobial activity	1 × 10^9^
*Bacillus subtilis* SK877	Silage	Digestive enzyme activity	1 × 10^8^
*Bacillus subtilis* BBG-B20	Chick fecal	Digestive enzyme activity	1 × 10^8^
*Saccharomyces cerevisiae* BBG-Y6	Soil	Digestive enzyme activity	1 × 10^8^

^1^ Korean traditional fermented cabbage. ^2^ cfu, colony forming unit.

**Table 2:** Ingredients and chemical compositions of the basal diet fed from 21 to 42 d of age.

Item	Basal Diet
Ingredients, %	
Ground corn	64.9
Soybean meal	20.0
Wheat bran	2.90
Tallow	3.87
Salt	0.12
Molasses	4.00
Tricalcium phosphorous	0.75
Lycine	0.36
Methionine	0.05
Choline chloride	0.05
Mineral premix ^1^	2.00
Vitamin premix ^2^	1.00
Total	100
Energy value and chemical composition	
Digestible energy, kcal/kg	3500
Dry matter (DM), %	86.8
Crude protein, % DM	21.2
Ether extract, % DM	7.37
Crude ash, % DM	5.17
Calcium, % DM	0.65
Phosphorus, % DM	0.80

^1^ Containing per kg: Fe, 100 mg; Cu, 50 mg; Zn, 25 mg; Mn, 15 mg; Co, 2.5 mg; I, 0.1 mg. ^2^ Containing per kg: vitamin A, 25,000 IU; vitamin D3, 5000 IU; vitamin E, 30 mg; thiamin, 1.0 mg; riboflavin, 15 mg; vitamin B6, 2.5 mg; niacin, 75 mg.

**Table 3:** The actual microbial counts of the additives before being fed to the animals.

Item	log10 cfu/g
Lactic acid bacteria	8.30
*Bacillus*	8.94
Yeast	8.59

**Table 4:** Effects of microbial-additive supplementation on the growth performances of weaning pigs fed for 21 d.

Item ^1^	Supplementation Levels, % ^2^				SEM ^3^	Contrast ^4^		
	0	0.5	1.0	1.5		L	Q	C
Initial body weight, kg	7.93	7.40	7.72	7.74	1.609	0.817	0.906	0.767
Final body weight, kg	14.2	13.7	14.5	14.6	2.177	0.966	0.974	0.692
ADFI, kg/d	0.44	0.43	0.44	0.47	0.043	0.960	0.440	0.404
ADG, kg/d	0.30	0.30	0.32	0.33	0.027	0.428	0.650	0.504
Feed efficiency	0.68 ^b^	0.69 ^b^	0.73 ^a^	0.70 ^b^	0.020	0.069	0.681	0.856

^a,b^ Means in the same row with different superscripts differed significantly (*p* < 0.05). ^1^ ADFI, average daily feed intake; ADG, average daily gain. ^2^ Supplementation of microbial additives in the basal diet at 0, 0.5, 1.0, and 1.5% of the feed. ^3^ SEM, standard error of mean. ^4^ L, linear effect; Q, quadratic effect; C, cubic effect. Significance of contrast was declared at *p* ≤ 0.05, while tendency of contrast was considered at 0.05 ≤ *p* ≤ 0.10.

**Table 5:** Effects of microbial-additive supplementation on blood metabolites of weaning pigs fed for 21 d.

Item ^1^	Supplementation Levels, % ^2^				SEM ^3^	Contrast ^4^		
	0	0.5	1.0	1.5		L	Q	C
IgG, mg/dL	633.7	640.6	682.0	685.8	96.48	0.024	0.966	0.662
Insulin, μU/mL	0.28 ^b^	0.42 ^b^	0.64 ^a^	0.46 ^b^	0.177	0.650	0.034	0.270
IGF-1 ng/mL	75.9	84.7	86.2	79.4	11.24	0.595	0.043	0.625
BUN, mg/dL	9.64	9.96	10.6	10.1	2.404	0.057	0.423	0.881
Blood glucose, mg/dL	99.9 ^b^	103.8 ^b^	123.2 ^a^	105.9 ^b^	10.52	0.086	0.001	0.140

^a, b^ Means in the same row with different superscripts differed significantly (*p* < 0.05). ^1^ IgG, immunoglobulin G; IGF-1, insulin-like growth factor 1; BUN, blood urea nitrogen. ^2^ Supplementation of microbial additives in the basal diet at 0, 0.5, 1.0, and 1.5% of the feed. ^3^ SEM, standard error of mean. ^4^ L, linear effect; Q, quadratic effect; C, cubic effect. Significance of contrast was declared at *p* ≤ 0.05, while tendency of contrast was considered at 0.05 ≤ *p* ≤ 0.10.

**Table 6:** Effects of microbial-additive supplementation on the fecal microflora of weaning pigs fed for 21 d (log10 cfu/g).

Item	Supplementation Level, % ^1^				SEM ^2^	Contrast ^3^		
	0	0.5	1.0	1.5		L	Q	C
Day 0								
Lactic acid bacteria	5.33	5.28	5.31	5.30	0.303	0.871	0.584	0.333
* Salmonella*	4.55	4.41	4.45	4.44	0.271	0.133	0.255	0.498
* E. coli*	4.01	3.94	3.88	3.93	0.468	0.448	0.702	0.480
Day 21								
Lactic acid bacteria	5.81	5.98	5.99	6.02	0.124	0.064	0.284	0.444
* Salmonella*	4.09	3.71	3.43	3.18	0.171	0.033	0.255	0.498
* E. coli*	3.86	3.74	3.18	3.33	0.468	0.048	0.702	0.480

^1^ Supplementation of microbial additives in the basal diet at 0, 0.5, 1.0, and 1.5% of the feed. ^2^ SEM, standard error of mean. ^3^ L, linear effect; Q, quadratic effect; C, cubic effect. Significance of contrast was declared at *p* ≤ 0.05, while tendency of contrast was considered at 0.05 ≤ *p* ≤ 0.10.

## Data Availability

Data are available on request from the corresponding author with reasonable reason.
